# Modeling the Leukemia Microenviroment *In Vitro*

**DOI:** 10.3389/fonc.2020.607608

**Published:** 2020-12-17

**Authors:** Cristina Scielzo, Paolo Ghia

**Affiliations:** ^1^ Unit of Malignant B Cell Biology and 3D Modeling, Division of Experimental Oncology, IRCCS Ospedale San Raffaele, Milano, Italy; ^2^ Unit of B Cell Neoplasia, Division of Experimental Oncology, IRCCS Ospedale San Raffaele, Milano, Italy; ^3^ Università Vita-Salute San Raffaele, Milano, Italy; ^4^ Strategic Research Program on CLL, Division of Experimental Oncology, IRCCS Ospedale San Raffaele, Milano, Italy

**Keywords:** chronic lymphocytic leukemia (CLL), B cell malignancies, microenvironment, *in vitro*, 3D models (three dimensional)

## Abstract

Over the last decade, the active role of the microenvironment in the pathogenesis, development and drug resistance of B cell malignancies has been clearly established. It is known that the tissue microenvironment promotes proliferation and drug resistance of leukemic cells suggesting that successful treatments of B cell malignancies must target the leukemic cells within these compartments. However, the cross-talk occurring between cancer cells and the tissue microenvironment still needs to be fully elucidated. In solid tumors, this lack of knowledge has led to the development of new and more complex *in vitro* models able to successfully mimic the *in vivo* settings, while only a few simplified models are available for haematological cancers, commonly relying only on the co-culture with stabilized stromal cells and/or the addition of limited cocktails of cytokines. Here, we will review the known cellular and molecular interactions occurring between monoclonal B lymphocytes and their tissue microenvironment and the current literature describing innovative *in vitro* models developed in particular to study chronic lymphocytic leukemia (CLL). We will also elaborate on the possibility to further improve such systems based on the current knowledge of the key molecules/signals present in the microenvironment. In particular, we think that future models should be developed as 3D culture systems with a higher level of cellular and molecular complexity, to replicate microenvironmental-induced signaling. We believe that innovative 3D-models may therefore improve the knowledge on pathogenic mechanisms leading to the dissemination and homing of leukemia cells and consequently the identification of therapeutic targets.

## Introduction

To date, a cure for cancer remains a major unmet clinical need and the possibility to achieve it relies on an increasing knowledge of the fundamental biological and molecular mechanisms leading to neoplastic transformation. In recent decades, novel experimental strategies have allowed for great advancements in cancer research providing major insights into the complexity of tumor development (1, 2). Particular attention has been dedicated to the design and implementation of experimental models that may allow for the study of human tumors in the setting of a research laboratory in a reproducible and consistent manner, through both the generation of tumors in living organisms (*in vivo* models) and the creation of culture systems of increasing complexity (*in vitro* models).

For many years, cancer pathophysiology studies and drug testing have relied on conventional 2-dimensional (2D) cell cultures *in vitro* systems and animal models (3). Although widely used, these models have a number of limitations thus poorly reflecting the *in vivo* situation and the actual responses to therapies. In particular, by definition, 2D cultures are lacking the physiological 3-dimensional (3D) structure of human tissues, whereby cell-cell and cell-extracellular matrix (ECM) interactions occur creating the so-called microenvironment. Not only the microenvironmental interactions but also the 3D structure itself are considered key for cell proliferation, differentiation and mobility, as they occur in the context of cancer development (4). In addition, animal models are expensive, time consuming and may not adequately reproduce the features of human tumors, present the correct immune activation or predict drug responses. *In vitro* 3D tissue models could provide a third approach that bridges the gap between traditional 2D culture and animal models (5). 3D cultures have obtained popularity in the study of solid tumor biology, being able to address several questions that are difficult to unravel by using conventional 2D culture models, such as in the event of metastasis and invasion, aggressiveness, dormancy and cell-cell interactions (6). In particular spheroids and organoids are the most established systems for different cancers. Spheroids represent the simplest model of 3D organization; as the name suggests, tumor cells, including primary cells, aggregate in spherical shapes. Organoids are more complex and are developed from embryonic induced-pluripotent and somatic stem cells and cancer cells or from primary tumor biopsy. The latter have the advantage of preserving the intact structure of the original tumor tissue along with its heterogeneity, morphology and gene pathways (7). These 3D models are widely used for solid tumor but it is now becoming clear that they may also be relevant for hematological cancers, in particular when assessing *in vitro* responses to drugs where 2D models poorly predict the actual clinical outcome (8).

Recently, *in vitro* 3D models mimicking specialized microenvironments of lymphoid tissues and incorporating advanced biomaterials and microfluidics, helped identify novel cellular, biochemical, and biophysical interactions and elucidate new regulatory mechanisms and potential therapeutic targets that could not otherwise have been studied in conventional 2D cultures (9). Several 3D systems have also been applied to the study of different B cell malignancies; however, this has only recently been used for CLL and with rather limited attempts.

**Figure 1:**
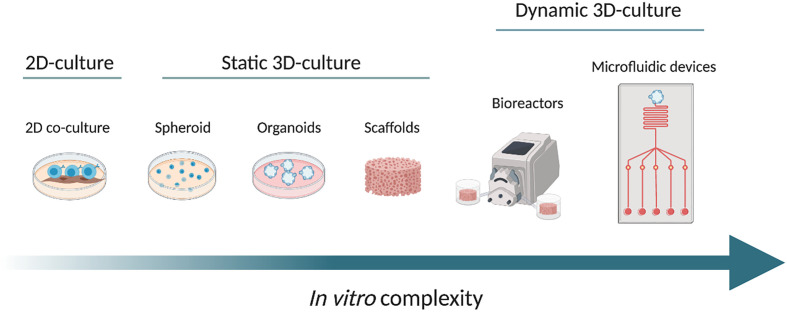
From left to right, the scheme shows the increasing complexity of the *in vitro* models, from a conventional 2D co-culture model (on the left), through different static 3D culture systems namely spheroid, organoids and scaffolds. On the right side, we propose examples of dynamic cultures: bioreactor and microfluidic device (at the far right). The arrow from left to right shows the increase in complexity. Created with BioRender.com.

In this review, we will discuss the design and propose of the use of new 3D *in vitro* models for B cell malignancies, in particular in the context of CLL research, discussing the key molecules/signals present in the tissue microenvironment that are likely needed to reliably replicate microenvironmental-induced signaling in such systems with increasing complexity *in vitro* (**Figure 1**).

### The Tissue Microenvironment in B Cell Malignancies

The specialized microenvironments of lymphoid tissues affect immune cell function and play an important role in the pathogenesis of hematological cancer by providing protective and supportive niches for malignant cells as paradigmatically shown in the case of chronic lymphocytic leukemia (CLL). Signals occurring in tissue compartments (Bone Marrow - BM) and secondary lymphoid organs are pivotal for survival, proliferation, homing and trafficking of the malignant cells (10). Calissano et al., by using *in vivo* deuterium labelling as an indicator of cells that had divided, documented the dynamic cellular kinetics and intraclonal complexity of CLL cells (11). These studies demonstrate that CLL is a dynamic disease where cells traffic and home between the peripheral blood (PB), bone marrow (BM), and the lymphoid tissues and here proliferate and die at variable rates. In particular, with the same deuterium labelling method, Herndon et al. identified the lymph node (LN) as the anatomical site where the majority of the proliferation occurs in contrast to the BM (12). In tissues, CLL cells make contacts with various types of cells, such as non-malignant stromal cells, nurse-like cells (or lymphoma associated macrophages), T lymphocytes, and mesenchymal-derived stromal cells. There, cells are also exposed to integrins, chemokines, cytokines and other survival factors (13). In particular, the LN microenvironment promotes B-cell receptor signaling, NF-kB activation leading to CLL cells proliferation (14). One has to note that only a small proportion of CLL cells proliferate in the so-called proliferation centers within the LN while the vast majority are resting or anergic (15) thus suggesting a very specialized and segregated structural organization within the tissues. For all these reasons, it is very challenging to reproduce *in vitro* what occurs *in vivo* possibly leading to CLL cells proliferation. Recently, Haselager et al. gave an overview of *in vivo* versus *in vitro* signals involved in CLL cells proliferation that we should take into consideration for the development of more complex *in vitro* models (16).

### Mimicking the Microenvironment: A Story of Increasing Complexity

It is then clear that the complexity of the interactions occurring *in vivo* and the cell heterogeneity present in the leukemic microenvironment pose a number of challenges when trying to dissect the specific role of each component in the development of CLL. Based on this, it becomes obvious that traditional preclinical *in vitro* modeling using only primary CLL cells or even cell lines have become obsolete. This is clearly evident by the fact that CLL cells undergo spontaneous apoptosis when cultured *in vitro* unless substitutes of survival signals found in the tumor microenvironment are provided (17). It is also interesting to note that many novel therapeutic agents currently under development for CLL are targeting not only intrinsic CLL signaling pathways, but also key CLL-microenvironment interactions. Because of this, traditional read-outs, such as *in vitro* cell death, are therefore limited and unable to fully evaluate these agents and unravel novel mechanisms of action. For example, the low rate of cell apoptosis induced *in vitro* by the BTK inhibitor ibrutinib would not have allowed for the identification of this compound as a revolutionary therapeutic agent through a traditional *in vitro* drug screening approach (18).

With this in mind, a series of improvements in 2D cultures have been implemented throughout the years by adding progressively more microenvironmental components aiming at improved reproducibility of the *in vivo* complexity and function.

### The Cellular and Molecular Components of the Tissue Microenvironment

Describing the components present in the tissue microenvironment is not the focus of this review [recently reviewed by Haselager et al. (16)] and we will provide a brief overview on few components that may have more relevance when considering building a functional system *in vitro*.

Nurse-like cells (NLCs) are found in secondary lymphoid organs where they activate the BCR signaling in CLL cells and secrete CXCL12 and CXCL13 that attract CLL cells into the tissue microenvironment. They also express BAFF and APRIL that promote survival and proliferation of CLL cells, thus providing full support for the leukemic cells as seen *in vitro*. Similarly, BM stromal cells (BMSCs), which are crucial for the well-being of normal haematopoietic cells, also regulate the survival of malignant cells in the bone marrow. The interaction between VCAM-1 on the BMSCs and VLA-4 integrin on CLL cells leads to the upregulation of the anti-apoptotic molecule MCL1. In addition, BMSCs, similarly to NLCs, secrete CXCL12 that interacts with CXCR4 on CLL cells, promoting tissue homing and regulating cell trafficking (19, 20). Keep in mind that FDCs and endothelial cells play a non-redundant role for tissue homing and CLL retention in tissues as well (21). In particular, adhesion to microvascular endothelial cells promotes CLL cells survival, activation and drug resistance along with two neuroendocrine secretory polypeptides that enhance the endothelial barrier function, for instance chromogranin A (CgA) and its N-terminal fragment (called vasostatin-1, CgA1-76), which circulate in variable amounts in the blood of patients with CLL (22).

**Figure 2:**
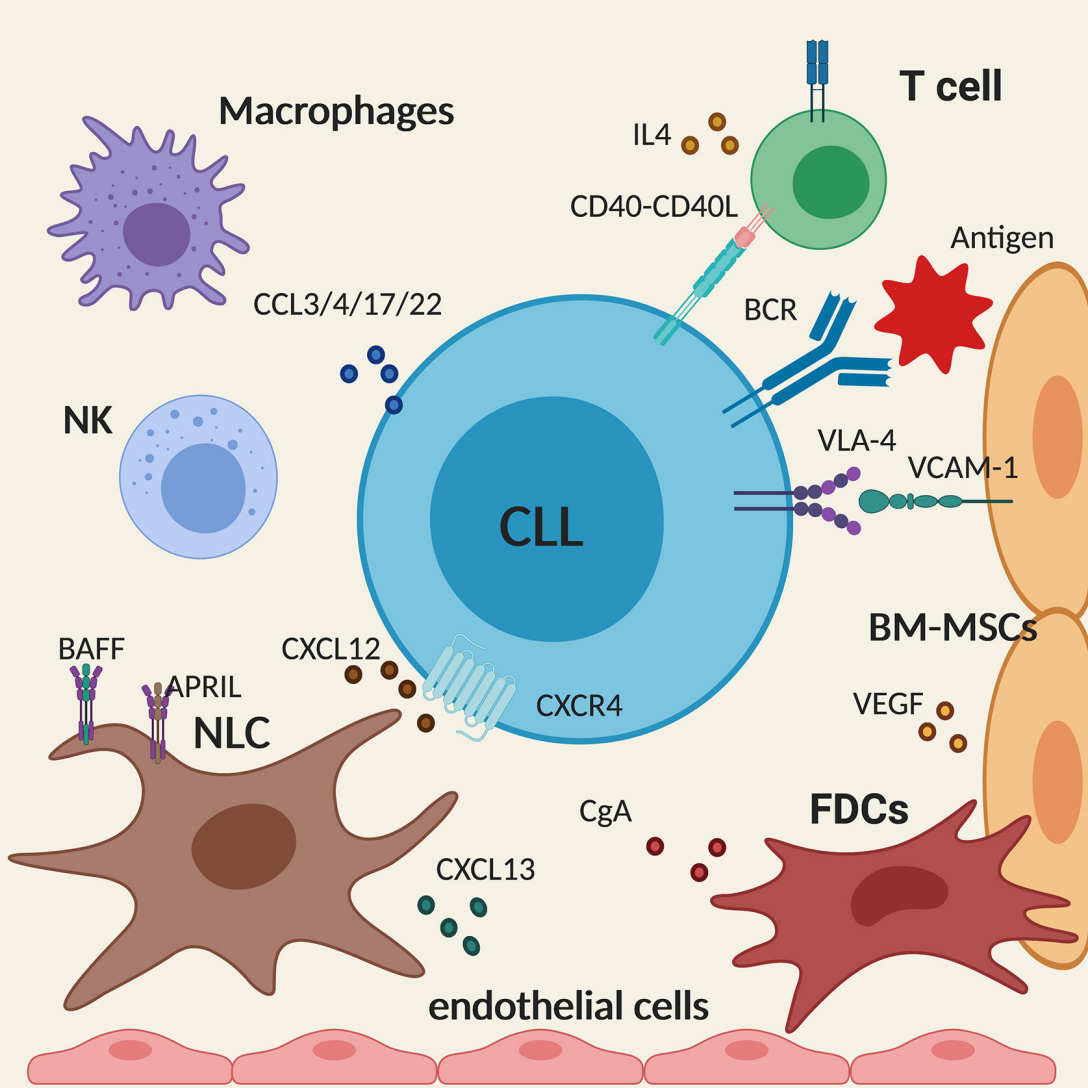
Schematic summary of the main cellular and molecular components of the tissue microenvironment in chronic lymphocytic leukemia (CLL). The figure shows a CLL cell (in the centre) interacting with the different components of the microenvironment described in the text. Created with BioRender.com.

Activation of malignant B cells through CD40 and by IL4 secreted by CD4^+^ T cells also promote CLL survival in lymphoid tissues (23). Activated CLL cells secrete CCL3, CCL4, CCL17, and CCL22 that recruit T cells and monocyte/macrophages to the tissue sites allowing the interaction with the leukemic cells (24). Although playing a pro-tumor effect, it is interesting to note that, in patients with CLL, despite an increased number of circulating CD4^+^ and CD8^+^ T cells, their functionality is compromised as T cells fail to form immune synapses (25) thus providing an explanation for the diminished immune surveillance. Similarly, NK cells have a defective cytotoxic activity in CLL, due to the overexpression of HLA-G in the plasma of CLL patients that impairs NK cytotoxicity and induces NK apoptosis (**Figure 2**) (26).

### Cell Cocktails: Combination of Chronic Lymphocytic Leukemia Cells and Microenvironmental Elements

Over the last two decades, accumulated experimental evidence demonstrated that bone marrow mesenchymal stromal cells (BM-MSCs) in general can protect different types of leukemia from spontaneous and chemotherapy-induced apoptosis (27).

In the case of CLL, the most relevant, and indeed the most utilized cells in co-culture systems, are stromal and nurse-like cell (NLC). Stromal co-culture systems were first described by Panayiotidis et al. in 1996. They showed that culturing CLL cells in contact with BMSCs could increase the percentage of viable cells after 10 days of culture by more than 30% compared to controls. Similarly, CLL cells incubated *in vitro* with FDCs were protected from spontaneous apoptosis as a result of the ligation of CD44 on CLL cells and up-regulation of MCL1, a member of the BCL2 family of anti-apoptotic proteins (28). Later, the same positive effect in terms of leukemic cell survival *in vitro* has been demonstrated with different BM stromal cells (29). In other instances, in an attempt to simplify the culture systems and avoid the use of different types of cells, investigators have tried to replace the pro-survival effect delivered by stromal cells with single or multiple factors thought to be relevant. Among many tested, the most utilized have been: CXCL12, CD40L, and IL-4, to prolong CLL cell survival (30); anti-IgM to increase BCR and NF-kB signaling and overall activation of the leukemic cells; CpG (with or without IL-2) to induce proliferation besides prolonging survival (31).

As an evolution of the cell- or molecule-based approaches, more complex co-culture methods have recently been developed, however still in a 2D setting. Primo et al. (32) developed a novel *ex vivo* short-term culture system to potentially predict the clinical response to therapeutics including novel inhibitors (in particular PI3Kδ and BTK inhibitors) by quantifying not only the pro-apoptotic effect of conventional drugs but also the anti-proliferative effect that are characteristically exerted by kinase inhibitors. After a thorough evaluation, alone and in combination, of several cell-extrinsic factors known to affect survival and growth of CLL cells, the authors proposed an optimized co-culture setting with sustained viability and proliferation of CLL cells, resembling what may occur within the infiltrate tissues *in vivo*. The combination includes: a cellular adherent component (HS5 - BM derived stromal cells), soluble factors such as CpG + IL2 and the addition of human serum, CLL plasma and erythrocyte fraction.

However, all these systems are *per se* limited in the possibility to fully reproduce the situation and the events occurring *in vivo* due to the intrinsic limitations of a flat, 2D culture.

While in solid tumor research, the need to develop *in vitro* tissue models with a 3D structure resembling *in vivo* tumor growth was more obvious, this need was perceived much later in hematological cancers, in particular CLL, due to the circulating nature of the disease. It is now evident that the circulating cells do not represent the actual reservoir of the disease, that is composed by the cells accumulating in solid tissues, in particular in the proliferation centers within LN or the BM (11, 23). For this reason, also a 2D culture system for CLL, regardless of its complexity and efficiency, needs to be implemented if we want to study the specific mechanisms of leukemic growth and invasion.

### 3D Culture Strategies

For all these aforementioned motivations, the proposed concept is that more complex culture systems, including 3 dimensional ones, should be explored in order to better mimic the physiological settings in which cells grow. Over the last few years, a number of 3D culture systems have been designed and developed and this has been evidenced by the increasing number of publications on this topic (33). The term 3D culture refers to a three-dimensional system in which cells are no longer cultured on a plastic or a glass surface but instead are allowed to proliferate, migrate, communicate and behave more physiologically.

### Disease Modeling in Static *In Vitro* 3D Cultures

In the broadest sense, 3D cell cultures can be categorized into two distinct types: scaffold-based or scaffold-free. Scaffold-based models support anchorage dependent growth and supporting structures are made of polymeric hard materials or hydrogels. In contrast, scaffold-free systems enable anchorage independence cell growth such us in the context of spheroid obtained by hanging drop microplates, magnetic levitation or ultra-low attachment coatings (33).

As an example of the latter models. Farinello et al. set up a scaffold-free co-culture to form a lymphoid aggregate to study murine CLL cells and stromal cells interactions by using collagen to promote the formation of so-called *spheroids*. In detail, by a hanging drop method they obtained polymerized stromal cells-collagen drops to which they added, after 24h of culture in petri dish, murine Eμ-TCL1 leukemic cells. The latter model is well known as it appears to reproduce human CLL in particular the most aggressive form, in terms of both biological and clinical features. Exploiting this system, the authors were able to validate a previously unknown mechanism regulating cell adhesion to stroma, through the retinoid-signaling pathway (34). Setting a 3D model with murine leukemic cells is uncommon but may also provide additional advantages allowing to compare more easily cells from different compartments of a living body, to produce a higher number of replicates from individual animals while limiting the number of utilize mice and to preserve human cells (in particular from tissues) for conclusive experiments for when preliminary results have been obtained with the mouse cells. Obvious criticism to this approach lies on the potential relevant differences in terms of functional behavior of mouse leukemic cells as compared to human CLL.

In terms of scaffold-based 3D cell culture systems, the most common models can be further categorized as either biological or synthetic. The former has the advantage of containing extracellular matrix (ECM) components (e.g., sugars, amino acids, lipids, proteins) thereby more closely resembling the actual tumor microenvironment (“biomimicry of the tissue of origin”). Examples of this model include Matrigel and Collagens or decellularized tissues or organs. However, natural products have innate differences during the commercial production process leading to heterogeneity in their composition between different lots, also based on the tissues of origin, thus impeding full potential reproducibility of the experimental settings.

On the other hand, synthetic or animal-free polymers scaffolds are made of biologically compatible polymers and hydrogels (35, 36) that do not exist in the tissues but have the advantage of being highly reproducible and also provide low cost, consistent and tunable scaffolds. Examples include: gelatin, cellulose, chitosan, alginate, recombinant silk, PLA (polylactide), and PCL (polycaprolactone) (37). In this case, nutrients and other factors can be added as needed and ideally cells cultured in the scaffold could produce their own functional ECM.

Both scaffold types have been used to recreate lymphoid tissues. The BM microenvironment niche has been the most investigated for acute myeloid leukemia (AML) and multiple myeloma (MM) to study the resistance to chemotherapeutics. Aljitawi et al. designed a 3D BM-microenvironment by co-culturing AML cell lines with human bone marrow derived mesenchymal cells in a synthetic scaffold and demonstrated significant differences in leukemic cell response to chemotherapy. In particular, leukemic cells cultured in 3D were more resistant to drug-induced apoptosis compared to cells cultured in 2D (38). De la Puente et al. adopted a different approach for MM by using a biological 3D scaffold from the BM of patients. Fibrinogen present in the plasma of the supernatant of the BM was cross-linked with calcium and this process allowed the encapsulation of MM cells, stromal cells, and endothelial cells. They demonstrated that this model could mimic the native MM growth and interaction with the microenvironment as well as drug availability (39). Notably, also in the case of MM, cells grown in 3D cultures showed an increased resistance to chemotherapeutic agents thus resembling more closely the *in vivo* response to therapy.

A variant of the scaffold-based models are 3D tumor organoids, that are *in vitro* 3D cellular clusters directly derived from primary tissues, embryonic stem cells, or induced pluripotent stem cells grown on artificial ECM (e.g., matrigel). Organoids exhibit similar organ functionality as the tissue of origin, and they are widely utilized for solid tumors and drug screening (40).

Tian et al. developed a type of 3D organoid system mimicking a lymphoid tissue to culture B and T cell lymphoma cell lines by using a functionalized hydrogel that allows one to precisely define the integrin density to be tested and incorporate FDCs as supporting stroma cell subtype (41). In this way, they recreated a condition that recapitulated the natural environment of these lymphomas and was suitable for drug testing.

### Innovative Dynamic In Vitro 3D Culture Models

Despite the improvements, 3D models have limitations due to the static condition of the culture system that cannot reproduce the dynamic interactions occurring *in vivo*. They are still lacking the possibility to study *ex vivo* CLL cells in a dynamic fashion with the possibility to study phenotypic or functional changes not only in time but also in space, during trafficking and homing to and from different tissue microenvironments.

*In vivo* cell growth is influenced by gravity, different flow regimes, shear and mechanical stresses. In addition, cells need an appropriate and continuous oxygen and nutrient transport to really mimic the *in vivo* situation. To this end, to have a functional vasculature system becomes crucial when planning the design of a viable 3D dynamic model. Thus, an additional level of complexity is needed to recreate the natural physiology of the tissue environment through the addition of a dynamic component to the *in vitro* system.

An example has been reported by Walsby and collaborators that developed an interesting *in vitro* dynamic system that modeled circulation and allowed for detailed study of transendothelial extravasation and migration of CLL cells (42). Thanks to this model, Pasikowska et al. (43), compared the migration capacity of LN derived CLL cells with those obtained from the peripheral blood (PB) and were able to show that the latter are constitutionally primed for lymphoid tissues homing and interaction with T cells.

Achieving this further step of complexity is feasible by using bioreactors or microfluidic devices. Bioreactors are closed systems in which biological and biochemical processes develop under monitored and controlled environmental and operational conditions, such as: temperature, pH, pressure, nutrient supply, and waste removal. Precise sensors inside the bioreactor connected to control software or pumps monitor the influx and efflux of nutrients and metabolites. Mass transfer regulation is critical since the shear stress may damage the cells and must be evaluated for each 3D model and cell type (44). Bioreactors have an important role in the *ex vivo* engineering of 3D tissues based on cells and scaffolds, including cell seeding of porous scaffolds, nutrition of cells in the construct and mechanical stimulation of the developing tissues. There are different types of bioreactors, among which: 1) spinner-flask bioreactors: used for the seeding of cells into 3D scaffolds and for subsequent culture of the constructs; 2) Rotating-wall vessels (RWV) bioreactors: it provides a dynamic laminar flow generated by a rotating fluid environment and it reduces diffusional limitations of nutrients and wastes, while producing low levels of shear flow; 3) direct perfusion bioreactors: here the culture medium is perfused directly through the pores of the cell-seeded 3D scaffold. During seeding, cells are transported directly into the scaffold pores, resulting in a uniform cell distribution (44). There are some examples of 3D culture coupled with dynamic growth in the case of B cell malignancies. To study MM, Belloni et al. (45) exploited a 3D Rotary Cell Culture System bioreactor using gelatin scaffolds. This particular type of bioreactor provides a balance between increased mass transfer and reduced shear stress, thus generating optimal conditions for long term *ex vivo* maintenance of tissue explants. This model was initially validated using a co-culture system where MM cell lines were placed in contact with stromal and endothelial cells. Next, the same system was then successfully applied to primary co-cultures of MM cells and BM stromal cells from patients together with endothelial cells (Huvec cell line), allowing the development of functional myeloma-stroma interactions and MM cell long-term survival and a more precise determination of the impact of the proteasome-inhibitor bortezomib on MM cells and on the microenvironment to predict actual responses. We recently adapted this model to study the interactions that CLL cells may engage with the BM tissue microenvironment (46). We co-cultured primary CLL cells and BM derived stromal cells on gelatin scaffolds maintained in the Rotary Cell Culture System bioreactor. This allowed for the parallel analysis of both CLL cells retained inside the scaffold (i.e., an environment that mimics the cells resident in the tissues) and those released in the external environment of the scaffold (to mimic circulating cells) in the presence or the absence of pharmacological agents. In particular, we used a BTK inhibitor, ibrutinib, that has the effect of mobilizing leukemic cells from the tissues, an effect that cannot be otherwise assessed and studied in a traditional 2D culture system. Thanks to the optimization of this model, we first observed that not all CLL cells are mobilized with the same efficiency from the scaffold; in particular primary CLL cells that express the protein HS1 in its inactive form (47) more frequently remain inside the scaffold. This somehow parallels an observation made *in vivo*, where patients treated with the kinase inhibitor show increased lymphocyte count due to mobilization of the leukemic cells from the tissues into the PB, thus underlining that this model may reliably reflect the *in vivo* situation. In addition to bioreactors, microfluidic techniques can also be used where 3D structures are connected to microchannels, made of polymers, to achieve a spatial control over nutrients and fluids (48). Microfluidics can be applied to different cancer models including hematological cancer [e.g., Acute lymphoblastic leukemia (49)], for phenotypic screening and personalized medicine. Many models recently developed include vasculature components that allow angiogenesis and migration (48). Interestingly, Cancer-on-chip models for lymphoid malignancies have recently been developed for drug discovery and mechanistic studies: a lymphoma-on-chip model for diffuse large B-cell lymphoma (DLBCL) was obtained by seeding tumor cells in a vascularized hyaluronic acid hydrogel placed on a chip and used to study the cross-talk between tumor cells, immune cells and endothelial cells and the response to drug treatment (50). These models serve also as the basis for the creation a LN-like structure. The most elaborate prototype of human artificial lymph node currently available was realized thanks to a miniaturized, membrane-based perfusion bioreactor, hosting a hydrogel matrix preloaded with dendritic cells through which T and B lymphocytes recirculated continuously and a set of microporous hollow fibres provided nutrient and gas exchange (51).

## Perspective/Discussion

Here, we reviewed *in vitro* models as crucial tools to study the pathophysiology of lymphoid malignancies, focusing in particular on the importance of exploring alternative models to study leukemia B cells in their tissue microenvironment. We believe that innovative 3D-models will be essential to improve our knowledge on the pathogenic mechanisms leading to the homing and dissemination of leukemia cells and consequently the identification of potentially important therapeutic targets. In our view, it is fundamental to combine cell growth in 3D with a dynamic system in order to obtain a more sophisticated and more biomimetic preclinical cancer model and 3D models combined with dynamic culture techniques show a great potential to accurately emulate the tumor microenviroment. Last but not least, it is necessary to develop suitable computational models (52, 53), to explore and predict in advance the best culture system for our disease modeling, the reason being the numerous variables that one has to consider by scaling up into a complex 3D co-culture dynamic *in vitro* system.

To conclude, we are just at the dawn of the next era where 3D culture system will become an indispensable tool for research and drug response assessment in B cell malignancies including CLL. For the latter, the 3D model in bioreactors is just the first step on a long path towards more complex 3D *in vitro* models that can better reproduce the interactions between leukemic cells and the tissue microenvironment in a dynamic fashion. That goes along with a deeper knowledge of the different cellular and molecular components that are fundamental to mimic the *in vivo* situation that will then become essential components of a more physiological structure that one day could even be envisioned to replace, at least in part, animal models. One has also to consider the specificities of the different microenvironments through which CLL cells recirculate implying the design and set-up of tissue-specific models that could eventually be connected to fully assess *in vitro* the complexity of the dynamic interactions occurring *in vivo*. The final goal will be to generate a 3D multi-organ system that could represent a new and versatile tool to understand leukemic cells behavior but also to test the specific effects of novel drugs or target therapies before embarking in complicated and expensive clinical trials.

## Author Contributions

CS and PG wrote the manuscript. All authors contributed to the article and approved the submitted version.

## Funding

The research leading to these results has received funding from AIRC under IG 2018 - ID. 21332 project and EHA advances research grant 2020 – P.I. CS. The research leading to these results has received funding from Fondazione AIRC under 5 per Mille 2018 - ID. 21198 program – P.I. Foà Roberto, G.L. PG; ERA NET TRANSCAN-2 Joint Transnational Call for Proposals: JTC 2016 (project #179 NOVEL), project code (MIS) 5041673 (to PG).

## Conflict of Interest

The authors declare that the research was conducted in the absence of any commercial or financial relationships that could be construed as a potential conflict of interest.

The reviewer YH declared a past co-authorship with one of the authors PG to the handling editor.
